# Mortality due to traffic accidents in Colombia: Profiles of pedestrians and cyclists, 1998-2019

**DOI:** 10.12688/f1000research.131431.2

**Published:** 2023-07-25

**Authors:** Gino Montenegro-Martínez, Maite-Catalina Agudelo-Cifuentes, Diana-Isabel Muñoz-Rodriguez

**Affiliations:** 1 Graduate School, Universidad CES, Medellín, 050021, Colombia; 2 Faculty of Nursing, Universidad CES, Medellín, 050021, Colombia; 3 Faculty of Physiotherapy, Universidad CES, Medellín, 050021, Colombia

**Keywords:** Traffic accident, mortality, pedestrian, cyclist, Multiple Correspondence Analysis, Road Traffic Injury

## Abstract

**Background**: Traffic accidents are an important issue for public health and a threat for sustainable development, with pedestrians and cyclists having been recognized as the most vulnerable actors on the streets. The objective of this study was to analyze the profiles of pedestrians and cyclists who died as a result of traffic accidents in Colombia during the 1998-2019 period.

**Methods: **An observational and descriptive study, with the deaths due to traffic accidents in Colombia between 1998 and 2019 as data source. Secondary data were taken from the Vital statistics of Colombia (EEVV), published by Departamento Administrativo Nacional de Estadística (DANE). A trend analysis of the number of deaths during the period under study was performed, and such number was examined against sex to identify potential differences. Multiple correspondence analysis was employed to elaborate the profile of pedestrians and cyclists who die due to traffic accidents. Three profiles were prepared for each road actor: a global profile, one for 1998, and another for 2019.

**Results: **The mortality profiles are different for pedestrians and cyclists, and, in turn, there are also demographic, geographic, and socioeconomic conditions in each type of road actor, which determine higher mortality risks. High population density, younger age group in the cyclists and adults among the pedestrians, low schooling levels and absence of health insurance are suggested as key factors in these profiles. Related to sex, for men is not possible to establish a profile. Women's cases are commonly related to health insurance, age, and population density.

**Conclusions:** Several contextual and demographic characteristics in pedestrians and cyclists allow delimiting mortality profiles. The profiles that were identified suggest the need to articulate road safety policies with other social and development policies in order to coordinate and integrate intersectoral actions that reduce mortality in these road actors.

## Introduction

Traffic accidents account for 50 million injuries and cause 1.3 million deaths across the globe. ^1^ It is estimated that 942 disability-adjusted life years are lost in the world, with a higher rate in low-income countries (1,068 for every 100,000 individuals) than in high-income countries (593 for every 100,000 people). ^2^ Low- and middle-income countries concentrate 90% of the mortality due to traffic accidents. ^3^ Due to the associated costs, it can push families into poverty or into facing significant psychological, physical, and social harm. ^4^ Thus, it has been pointed out that traffic accidents are an important public health issue, ^5^ as well as one of the threats to sustainable development. ^3^

Target 3.6 from the Sustainable Development Goals (SDGs) ^6^ set out to reduce mortality due to traffic accidents by 50% by 2020 across the globe ^6^; however, only a 15.40% reduction has been achieved between 2015 and 2019. ^7^ Therefore, the deadline to achieve this target, among others, has been extended to 2030, instructing the countries to implement intersectoral, integrated, and comprehensive interventions to attain safe and sustainable transportation. ^5^ The aforementioned implies that the road safety agenda should be linked to other political plans, such as children’s health, weather-related actions, gender, and equality, among others. ^3^

In Colombia, it has been estimated that the number of deaths due to traffic accidents between 2015 and 2021 was 47,916. It is the fourth most common cause of death among people aged from 15 to 49 years old, and it is estimated that its incidence implies 918 disability-adjusted life years lost. ^8^ The costs related to traffic accidents were around USD 815.5 million for 2016. ^9^

Given the behavior in terms of the number of traffic accident victims, motorcyclists, pedestrians, and cyclists have been recognized as the most vulnerable road actors. ^1^ One-third of the deaths in the country corresponds to pedestrians and cyclists: the number of deaths in the last five years has been reduced by 10.1% in the former but has presented a 29.1% increase in the case of cyclists during the same period. ^10^

In the context of the decade of action for road safety 2020-2031, member states of the United Nations have been instructed to strengthen research in this field in order to understand the nature of the problem, as well as to identify effective solutions and strategies in road safety. ^3^^,^^11^ Additionally, in Colombia, research in the road safety field has been acknowledged as the basis for formulating and implementing strategies to prevent and mitigate the impacts of traffic accidents. ^12^

In this sense, studying profiles of the road actors considered vulnerable provides information that allows for analyzing the phenomenon comprehensively and adapting road safety policies that, in addition to viewing road actors in their singularity, consider them in their full complexity.

To the present day, there is no knowledge about studies in Colombia addressing the analysis of mortality due to traffic accidents from this perspective. Therefore, the main objective of this study was to analyze the profiles of pedestrians and cyclists who died as a result of traffic accidents in Colombia during the 1998-2019 period.

## Methods

### Study design

An observational and descriptive study was conducted, focused on the deaths due to traffic accidents in Colombia between 1998 and 2019. Secondary data were taken from the Vital statistics of Colombia (EEVV, in Spanish), published by Departamento Administrativo Nacional de Estadística (DANE) (freely available online here). The STROBE guidelines (Strengthening the Reporting of Observational Studies in Epidemiology) were followed for the present study.

### Study setting and procedure

Only deaths among pedestrians and cyclists were considered. The records included were those in which the basic cause of death was recorded with the following ICD-10 codes: V010 to V099 for deaths among pedestrians; and V100 to V199 in the case of deaths among cyclists.

For the characterization, the death year and the municipality of occurrence were considered to elaborate the “population density” variable, adopting the number of inhabitants for 2010 (approximately half of the period under analysis) and the size of the municipality. The number of inhabitants for 2010 was taken from population projections published by Departamento Administrativo Nacional de Estadística (DANE), Municipal series of the population by area for the period 1985-2017 (freely available online here); the size of the municipality was taken from Municipios de Colombia (freely available online here). Subsequently, based on these data, the population density for each municipality was calculated as follows: population/km2. Subsequently, the quartiles were obtained considering the density of all the municipalities to classify them into municipalities with low, moderate, high, and very high population density based. This measure was calculated by the authors (freely available in online here). Other sociodemographic variables were also used, such as sex, age, schooling level, and health insurance regime. Four major groups were assembled for the “age” variable: group 1 (<25y); group 2 (25-44y); group 3 (45-59y); group 4(≥60y).

In Colombia, health services are provided through the affiliation of the population to the Social Security General System (Sistema General de Seguridad Social, SGSSS). In health, this is divided into three affiliation regimes which, among other elements, are related to the link of the individuals into the labor market and their ability to pay a fee monthly. ^13^ The subsidized regime includes people lacking formal employment and those who cannot pay a monthly fee. The contributory regime encompasses those with a job contract or who work autonomously and are able to pay a monthly fee. The special regime includes those who work in the armed forces or who teach in the public sector, among others. ^13^ In this study, the “affiliation regime” variable was dichotomized into two groups: subsidized and contributory. This latter incorporates the individuals in the special regime, as it represents less than 5% of the population enrolled in the SGSSS. ^14^

### Data analysis

We performed a trend analysis of the number of deaths during the period under study for road actors stratified by sex to identify potential trends. Absolute and relative frequency measures were used for the characterization, and they were analyzed for each road actor (pedestrians and cyclists separately). Subsequently, multiple correspondence analysis (HOMALS, homogeneity analysis through alternating least squares) was used to identify the profiles of pedestrians and cyclists who die in traffic accidents. This statistical technique allows the representation of the relationship between categories of different variables in perceptual space. The principal advantage is representing columns and rows in the same space; the dimensions are characteristics not observable that allow the objects to gather in a multidimensional space. The principal variable method was used for normalization. ^15^ The main syntaxis to develop Multiple Correspondence Analyses are freely available here.

The results are presented in the form of bidimensional graphs, where the distances between the points show the relationships between the categories, and the similar categories are indicated as close to each other. The first dimension contains most of the information. The percentage of information explained by each of the dimensions is represented by the eigenvalue. For each model, the variance magnitude value was obtained as an indicator of the importance degree of each dimension in the global solution.

In order to observe changes in the profile of deaths among pedestrians and cyclists throughout the period, three profiles were prepared, one for each road actor: a global profile, one for 1998, and another for 2019. The IBM SPSSS Statistics program, Windows version 24.0 (Licensed to *Universidad CES*) (IBM Corp, 2016) (RRID:SCR_002865), was used to process the data and prepare the graphs. ^33^

## Results

In the period from 1998 to 2019, there were 52,226 deaths due to traffic accidents in Colombia: 44,203 pedestrians and 8,023 cyclists. A 48% reduction in the number of deaths among pedestrians was observed in the period analyzed; the year with the highest number of deaths was 1998 (2,854) the one with the fewest was 2017 (1,431). The behavior was different for the deaths among cyclists, showing variations over time and noticing that the highest number of deaths was in 2001 (462) and the lowest in 2013 (294); in general, the increase was 24% when comparing the number of deaths between 1998 to 2019.

**Figure 1.**
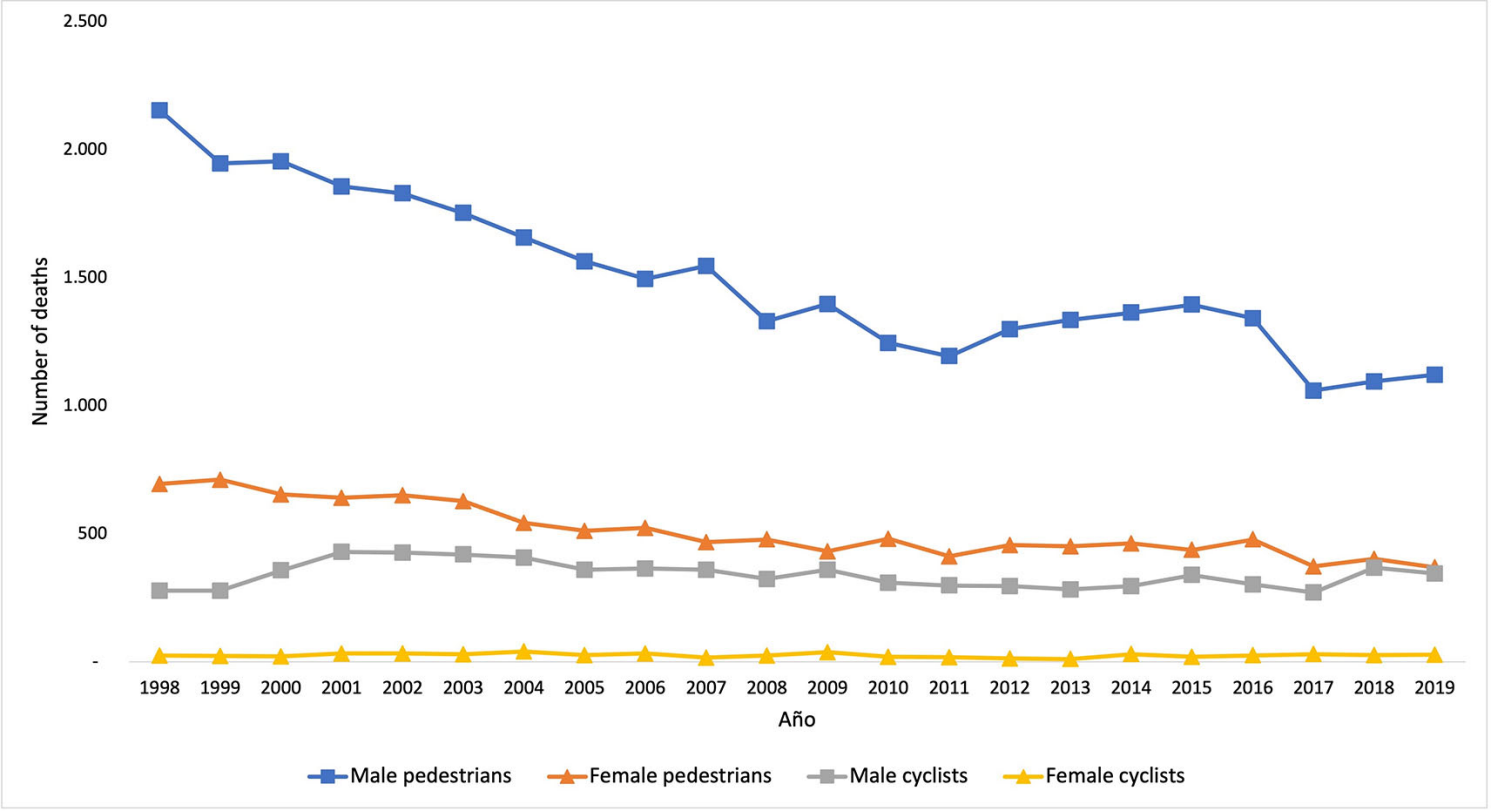
Trend corresponding to the number of deaths among pedestrians and cyclists between 1998 and 2019, according to sex.

The results corresponding to the death trend among pedestrians and cyclists in traffic accidents, both for men and women, are presented in Figure 1. The number of deaths among men was higher than among women, both for pedestrians and cyclists. The highest number of deaths among male pedestrians was recorded in 1998, whereas it was in 1999 for women; throughout the period, there were three deaths of male pedestrians for every death of a female one. Regarding cyclists, the highest numbers of deaths among men and women were recorded in 2001 and in 2004, respectively; the difference between sex was much higher, noticing that there were 13 deaths among men for every death of a woman throughout the period.

In 83.1% of the deaths, pedestrians were in municipalities with high population density. The highest proportion of deaths was among individuals aged over 60 years old, followed by people aged between 25 and 44 (22.3%). In terms of schooling, 52.7% of the deaths among pedestrians were recorded in people with Elementary School as their highest education level. 38.0% of pedestrians who died in traffic accidents were not enrolled in the health system.

On the other hand, most of the deaths among cyclists (88.3%) were in municipalities with very high population densities. Unlike what was observed in pedestrians, the proportion of deaths among cyclists was higher in individuals aged less than 25 years old; 53.5% of these deaths corresponded to people with Elementary School, and 35.6% were not enrolled in the health system ( Table 1).

**Table 1.** Sociodemographic characteristics of the pedestrians and cyclists who died in traffic accidents between 1998 and 2019 in Colombia.

	Pedestrians		Cyclists	
	N	%	N	%
**Sex**				
Male	32,938	74.5	7,465	93.0
Female	11,252	25.5	558	7.0
**Population density in the municipality of occurrence**				
Low	1,187	2.7	137	1.7
Moderate	2,254	5.1	294	3.7
High	4,048	9.2	510	6.4
Very high	36,714	83.1	7,082	88.3
**Age group**				
<25 years old	9,309	21.2	2,420	30.3
25-44 years old	9,754	22.3	2,122	26.5
45-59 years old	8,110	18.5	1,727	21.6
≥60 years old	16,654	38.0	1,724	21.6
**Schooling level**				
No schooling	3,793	16.5	285	6.0
Elementary School	12,092	52.7	2,563	53.5
High School	5,939	25.9	1,713	35.8
Higher Education	1,116	4.9	226	4.7
**Health insurance regime**				
Contributory	7,590	25.8	1,692	29.7
Subsidized	10,664	36.2	1,977	34.7
Uninsured	11,193	38.0	2,032	35.6

Related to population density, we observed that for each death of pedestrians and cyclists in cities with low, moderate, and high population density, approximately occurs five deaths in cities with very high population density (analysis is freely available here). According to the health insurance regime, the number of pedestrians and cyclists uninsurance has decreased by year; between 1998 to 2019, we report a decrease of 96.90% (are freely available here).

### Profile of the pedestrians who die due to traffic accidents in Colombia

Three important profiles that represent the main characteristics of the pedestrians who died due to traffic accidents were observed. A profile represented by the deaths in municipalities with low, moderate, and high population density, these cases corresponded mainly to people under 25 years of age. The deaths in municipalities with very high population density were mainly among individuals aged between 45 and 49 years old and were enrolled in the contributory health regime. On the other hand, the deaths among female pedestrians showed the affiliation to the subsidized health regime and Elementary School education as common elements. For males, it was not possible to establish a profile.

**Figure 2.**
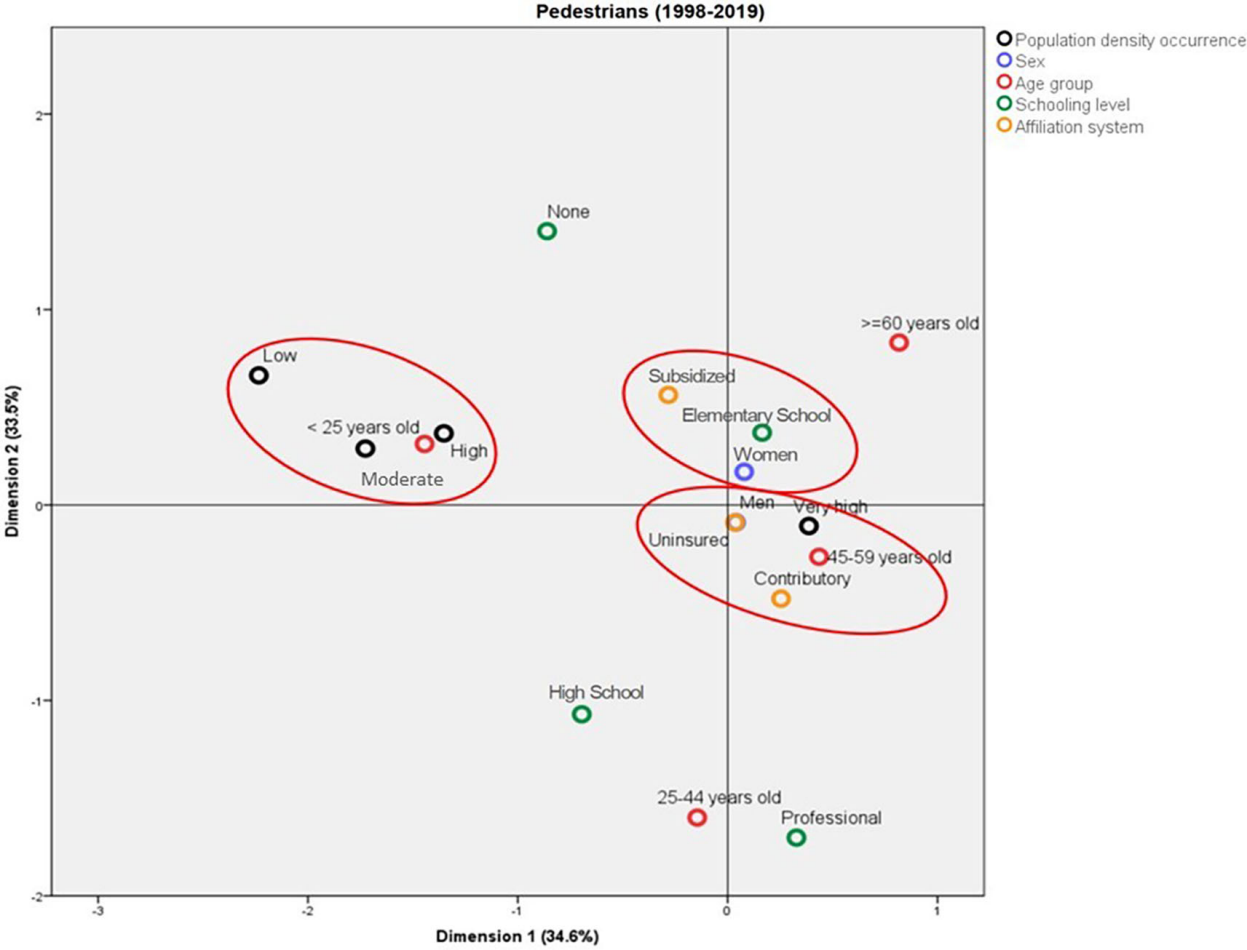
Profile of the pedestrians who died due to traffic accidents in Colombia between 1998 and 2019.

The profile of the deaths among pedestrians is shown in Figure 2; this model explains 68.1% of the variance, with Dimension 1 and Dimension 2 explaining 34.6% and 33.5%, respectively. The eigenvalues of both dimensions were relatively close to each other, which indicates that they have similar relevance for the model (5.89 and 5.69, respectively). According to the discrimination measures, the most important variable in Dimension 1 was “population density”, whereas it was “age group” for the second dimension. On the other hand, the variable that most contributed to explaining the total variance was “age group”, followed by “population density”.

**Figure 3.**
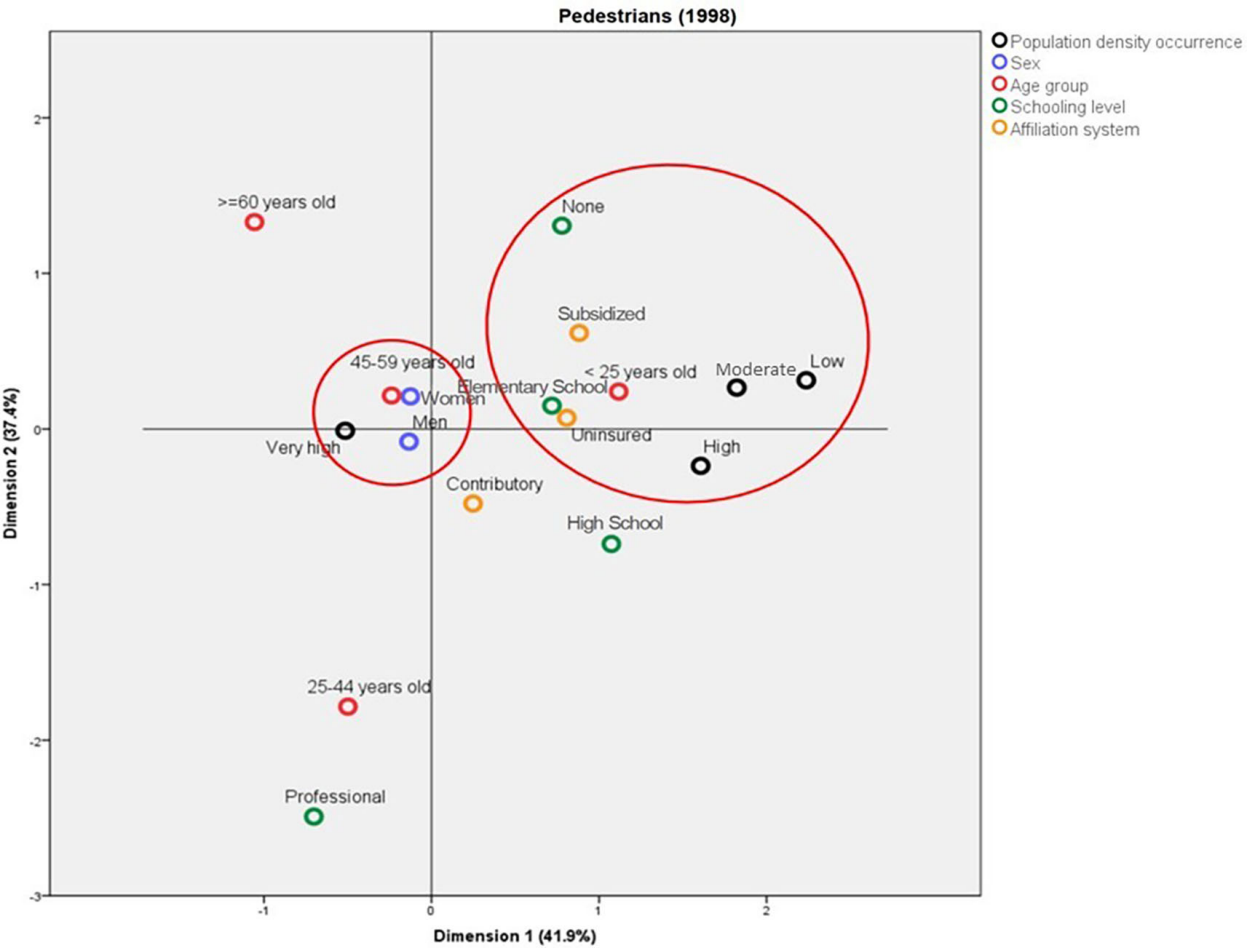
Profile of the pedestrians who died due to traffic accidents in Colombia during 2018.

**Figure 4.**
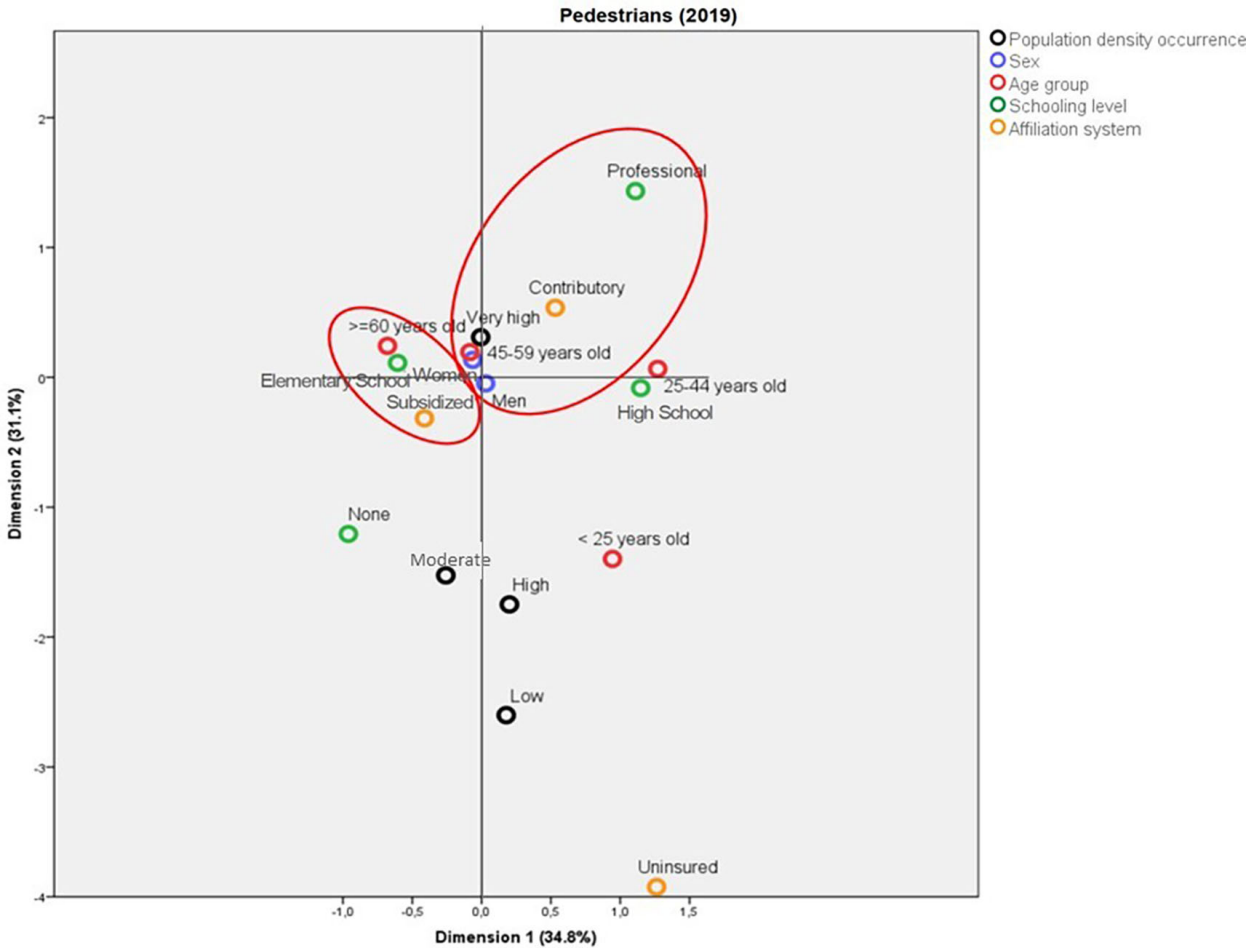
Profile of the pedestrians who died due to traffic accidents in Colombia during 2019.

The behavior of the deaths among pedestrians was compared between 1998 and 2019. For 1998, two profiles mainly differentiated by the population density of the municipality where the accidents happened and by the pedestrians’ age groups were identified; in this sense, the deaths in the municipalities with low and average population density were mainly in people aged less than 25 years old, with no schooling or with Elementary School as the highest level, affiliated to the subsidized regime or uninsured. On the other hand, the deaths in municipalities with very high population density were mainly in people aged between 45 and 59 years old ( Figure 3). In contrast, in 2019, the deaths in municipalities with very high population density corresponded to people enrolled in the contributory system and with a professional schooling level. Also in this year, the pedestrians aged more than 60 years old who died due to traffic accidents belonged mainly to the subsidized health regime and had Elementary School as their highest schooling level ( Figure 4).

### Profile of the cyclists who die due to traffic accidents in Colombia

Among the cyclists, the deaths due to traffic accidents differed mainly by age group. It is noticed that the deaths among cyclists aged less than 25 years old were characterized as females, with Elementary School as the highest schooling level. On the other hand, the cyclists aged between 25 and 44 years old had attained professional schooling levels, whereas the victims over 45 years old had Elementary School as their highest educational level. In contrast, the deaths in municipalities with low, moderate, and high population density corresponded to individuals that were illiterate and belonged to the subsidized affiliation regime.

**Figure 5.**
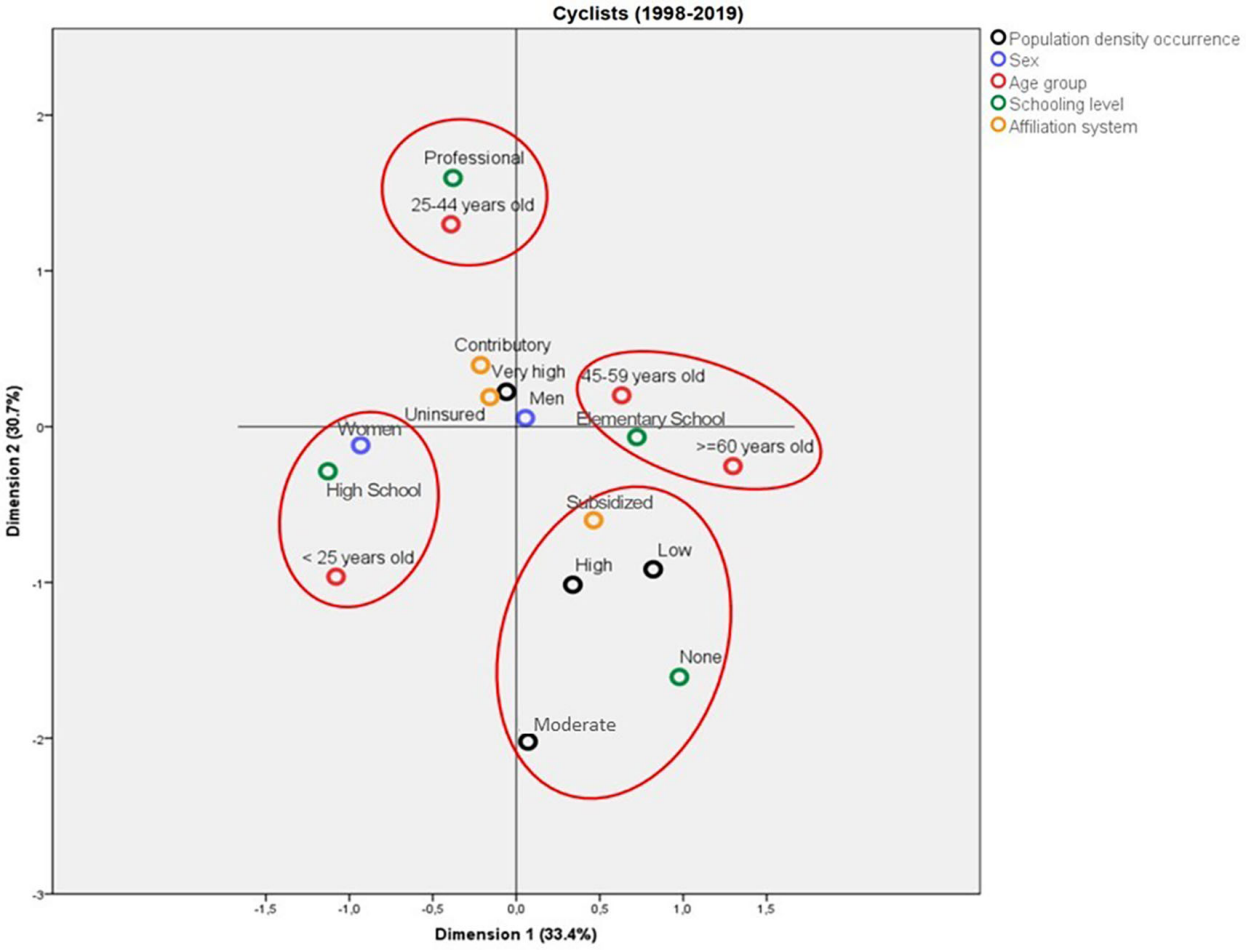
Profile of the cyclists who died due to traffic accidents in Colombia between 1998 and 2019.

The model obtained for the profile of the deaths among cyclists due to traffic accidents was able to explain a total variance of 60.5%, distributed in 31.1% explained by Dimension 1 and 29.4% by Dimension 2. The eigenvalues of both dimensions were relatively close to each other, which indicates that they have similar relevance for the model (5.92 and 5.58, respectively). According to the discrimination measures, it was observed that the most important variables for Dimension 1 were “schooling level” and “affiliation regime”, whereas it was “age group” for the second dimension. Sex and population density failed to discriminate well between both dimensions. On the other hand, the variable that most contributed to explaining the total variance was “age group” ( Figure 5).

**Figure 6.**
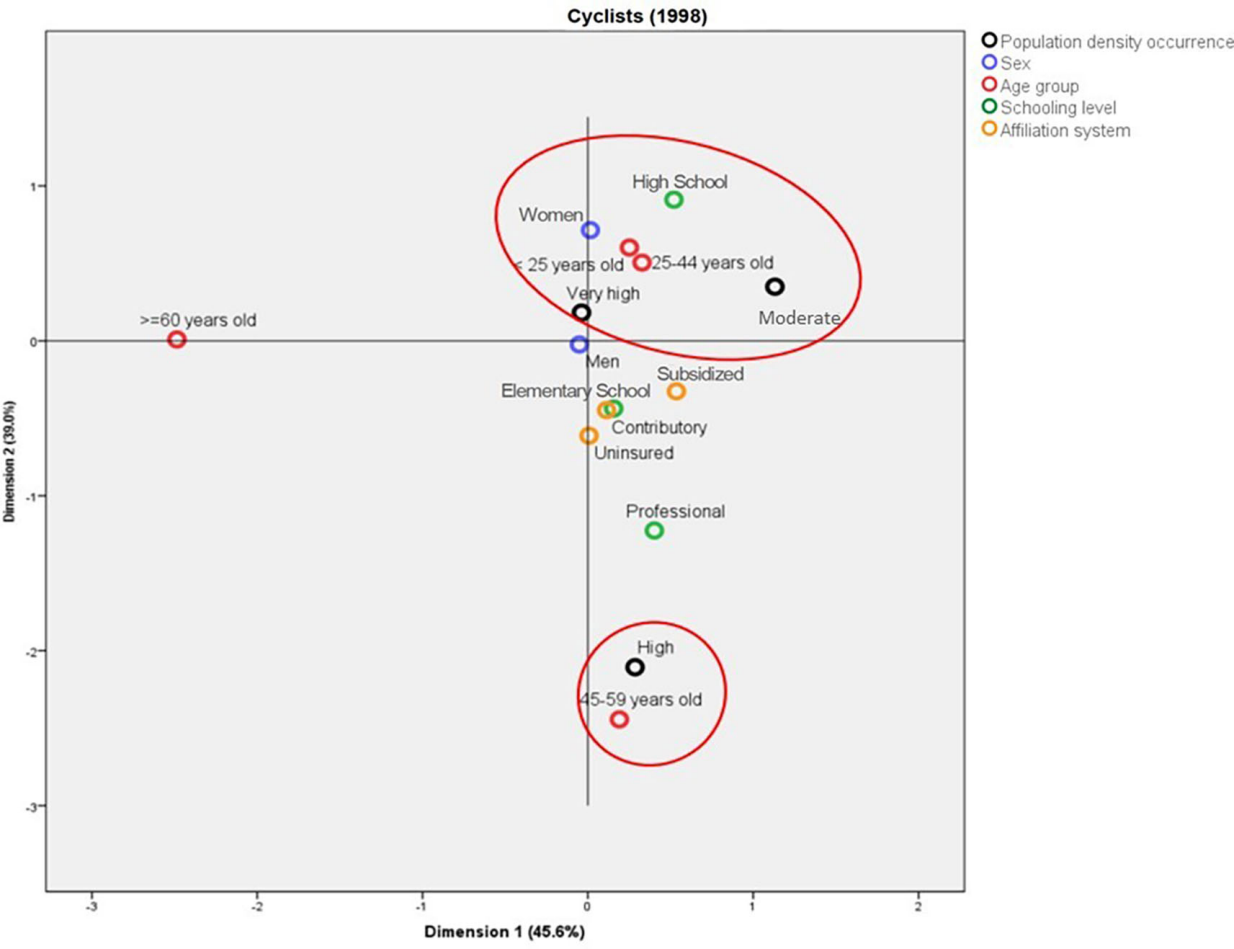
Profile of the cyclists who died due to traffic accidents in Colombia during 1998.

**Figure 7.**
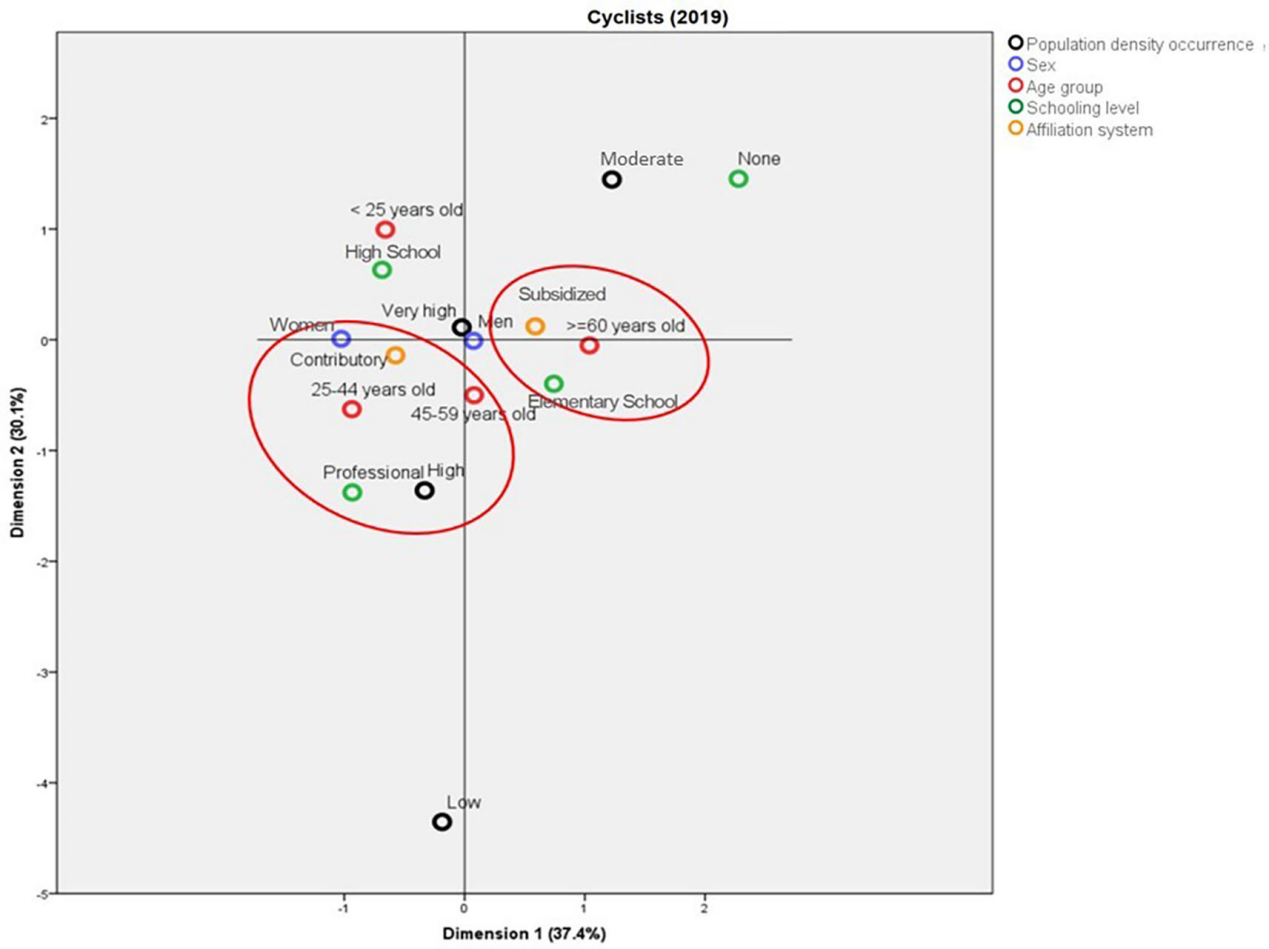
Profile of the cyclists who died due to traffic accidents in Colombia during 2019.

In 1998, the deaths among cyclists in municipalities with average population density were mainly in women aged less than 45 years old, with Elementary School as the highest schooling level. On the other hand, in the municipalities with high population density, these deaths were represented by people aged between 45 and 59 years old with professional studies ( Figure 6). In contrast with the above, for 2019, the deaths in municipalities with high population density were in people aged between 25 and 44 years old, with professional studies, from the contributory regime, and mainly women. In addition, it is possible to establish a profile for the cyclists aged more than 60 years old who die in traffic accidents: they mainly belong to the subsidized regime and have Elementary School as the highest schooling level ( Figure 7).

The deaths of male pedestrians and cyclists were variable. They did not reflect the presence of a specific profile that allows for determining the characteristics of the men that die on the streets due to traffic accidents.

## Discussion

The objective of this article was to analyze the profiles of pedestrians and cyclists who died as a result of traffic accidents in Colombia during the 1998-2019 period. Mortality was much higher among men than among women: three men for every woman in the case of the pedestrians and 13 men for every woman in the case of the cyclists. Despite the aforementioned, some profiles were identified that describe mortality in women; however, the variability of the characteristics of the men who die in traffic accidents did not allow for identifying a profile associated with men. Both for cyclists and pedestrians, the highest number of deaths was recorded in cities with high population density. 38.0% of pedestrians were over 60 years old, and, in the case of the cyclists, three out of ten were aged less than 25 years old. For both types of road actors, half of the victims had Elementary School as their highest level of schooling.

Although riding a bicycle and walking have been promoted as active transportation means to improve health and environmental conditions throughout the world, ^16^^,^^17^ there are few research studies focused on these road actors in low-middle countries, ^16^ perhaps due to the significant attention directed to the accidents involving motorcyclists, drivers and passengers of vehicles.

Particularly bicycles have been promoted as an alternative transport to mobilize into the cities and also as a strategy for sustainability. ^18^ This is the case for the principal cities in Colombia. In Bogotá, D.C., the country’s capital, has been rising by 16% between 2014 and 2017, the number of workers who mobilize through bicycle. Additionally, this has been accompanied by the building of infrastructure; in 2017, the inhabitants of Bogotá D.C. had 480 kms of bikeways (approximately 5.9 kms per 100,000 inhabitants), been higher than Cali and Medellín. ^19^ Considering the results of this research and the recent promotion of the bicycle as a means of transport, it is relevant to consider other safety aspects that allow the safe mobility of this road actor, in addition to infrastructure.

The higher mortality rate due to traffic accidents among men reported in this study is consistent with the global behavior ^20^ and showed no variation between the road actors. In the case of cyclists, it has been shown in other studies that men, mainly young ones, are a high-risk group related to greater exposure and lethality ^21^ as a result of risky behaviors such as rule infringements, distractions, and lack of control, ^22^ the person’s skill level, ^22^ riding a bicycle at night and without a helmet, and consumption of alcohol and psychoactive substances, ^23^ which are determinants for the accidents involving bicycles. ^24^

A comparative study of mortality due to traffic accidents showed that contrary to Spain and The United States, ^25^ Colombia has failed to achieve significant reductions and that there is a marked increase in mortality among men, mainly in those under 25 (an increase 17.6%). However, a reduction between 20% to 68% of mortality was evidenced among the women belonging to the same age groups. ^25^ This is congruent with our results; women had reductions in pedestrians by around 47% and in cyclists by 17%.

Particularly in our study, we found that the number of deaths among pedestrians has shown a reduction over time, almost by half, specifically in Colombia, although the reduction percentages have been higher in other countries, close to 60%. ^26^

We found the deaths among pedestrians are concentrated in older adults. In a study developed in Brazil, it has been described that they correspond to almost one-third of the overall mortality due to traffic accidents. On the other hand, pedestrians aged at least 60 years old present approximately 9.6 and 4.2 more risks of dying than people aged 0-19 and 20-59 years, respectively. ^27^

Among other reasons, the risk derives from the existence of a traffic environment that is essentially dangerous and challenging for pedestrians and cyclists. ^26^ Consequently, improving road safety for pedestrians is substantial, as it can be a representative element of the population’s quality of life. ^27^

In relation to the mortality profiles found, important differences were observed between municipalities with high, moderate, and low population density when compared to those with very high population density. Contrary to our findings, previous evidence has shown that population density has been inversely correlated with the number of deaths due to this cause. ^28^ This might be expected in areas where the interaction between vehicles and people would demand greater control, surveillance, and traffic signs to restrict the vehicles’ speed and protect pedestrians.

The common characteristics among the pedestrians who die in traffic accidents are mainly marked by age group and by the population density of the municipality where the accidents happen. However, they were also linked by schooling level, affiliation to the health system, and age group of the cyclists. In our study, we find that deaths by traffic accidents in pedestrians and cyclists are diverse and consider sociodemographic characteristics, similar to other studies. ^29^^–^^31^ Among others, it has been described that traffic accidents are more frequent in the lowest socioeconomic strata, as people belonging to these groups in the social hierarchy tend to indulge in more risky behaviors and because, on the other hand, their access to health services is more precarious. ^32^

Another factor associated with socioeconomic conditions is related affiliation to the health system. It has been shown that the delays in detecting the need to offer assistance and to provide care to traffic accident victims increase the severity of the injuries and, therefore, the probability of death. ^20^ Treatment of these traumas may demand critical time frames: a delay of only a few minutes can preclude saving a life. ^20^ In order to improve the care to be provided after the accidents, it is necessary to ensure that access to pre-hospital assistance is provided and to improve the quality of this care. ^20^

The spatial separation of the transportation means might improve people’s sensation of safety and prevent accidents and collisions. ^16^ Accident prevention might increase the willingness to walk and ride bicycles. ^16^ To such end, strategies that allow for improving road design, road education, and citizen culture should be adopted. It can be very useful to adapt to the local reality of successful experiences from countries such as Spain, which, although still reporting significant mortality rates, have shown effective strategies to reduce accidents and mortality due to this cause. ^16^

Regarding the socioeconomic conditions, many low- and middle-income countries lack policies to improve safety for pedestrians and cyclists, or such policies are not complied with (or enforced) by the various actors. ^28^ Especially in these countries, walking is a popular daily activity that offers extensive benefits for health and which, in addition, represents for many their only option to commute to the places they need for social functioning (schools, work, family, recreation). ^28^ Approximately 91 countries, 9.0% of them of high income, have policies to promote walking or riding bicycles ^28^; however, if these strategies are not accompanied by others, such as effective speed control and accessibility for pedestrians and cyclists, they might lead to an increase in the number of injuries due to traffic accidents. ^28^

A key strategy for a safe traffic system both for pedestrians and cyclists is to separate these users from the drivers of motor vehicles. ^27^ Other studies have also shown that reducing speed is more important than improving the design of the vehicles in order to decrease the severity of the pedestrians’ injuries; the existence of regulations and speed limit monitoring, and strict law enforcement are important to reduce the number of injuries among pedestrians and cyclists. More coordinated education in safety is required, combined with community safety promotion activities. ^28^

### Study limitations

One of the limitations while developing this study was the lack of information regarding other variables that are a fundamental component of this complex system to understand the factors influencing traffic accidents. For example, the probability of death can change according to the type of vehicle involved in the pedestrian’s or cyclist’s death; in addition, there are several differences in the collision factors and in the injury patterns between the collisions involving cyclists and pedestrians with and without motor vehicles.

However, the data reconstruction performed from 1998 to 2019 with essential variables in terms of inequalities is important, such as population density, sex, schooling level, and health insurance affiliation. This analysis and the profiles prepared to contribute to implementing evidence-based safety interventions. It has already been documented that these actions might prevent between 25% and 40% of all fatal injuries related to traffic accidents at the global level.

The main strength of this study is understanding differential mortality mechanisms for each road actor during an important period of time, including the characteristics (age, sex, Educational level, health insurance, and population density) of each one. The aforementioned becomes information for more reasonable decision-making processes when devising prevention strategies and, consequently, good results in reducing the mortality rates due to traffic accidents. This is in consequence of the challenges in road safety. ^4^

### Ethical considerations

This study was developed with an analysis of death data sets that are openly published and available online ( here).

In order to ensure data privacy, the records are anonymous. This study is the result of the research project: Mortality trends (1992-2017) due to road incidents in Colombia according to road actors: Educational inequities, rural/urban inequalities, a differential burden on life expectancy, and retrospective evaluation of public policies in cities, from the CES University and the Ministry of Science and Technology through call 844-2019. This project has the endorsement of the Institutional Human Research Ethics Committee of CES University (Act No. 172 of 2021).

## Authors’ contributions

Gino Montenegro-Martinez participated in conceptualization, formal analysis, Funding acquisition, investigation, methodology, project administration, supervision, the writing of the original draft, and the manuscript review & editing. Maite-Catalina Agudelo-Cifuentes participated in data curation, formal analysis, investigation, methodology, the writing of the original draft, and the manuscript review & editing. Diana-Isabel Muñoz-Rodriguez participated in formal analysis, investigation, writing of the original draft, and manuscript review & editing.

## Data Availability

No primary data are associated with this article. The secondary data used for this research, taken from the Vital statistics of Colombia, are freely available from the Departamento Administrativo Nacional de Estadística (DANE), freely available here. The data used for this study are available in the National Data File of Colombia. On this page, DANE makes available the anonymized open data from the different annual surveys carried out in the country. Users can access databases in specialized formats, such as SPSS, and general use formats, such as TXT, with many variables, which they can use unlimited. To access this data, you must enter this link, then go to “Citizen Service,” “Open data: microdata and macro data.” In the “Society” menu, select “Demographics and population” and enter the “EEVV Vital Statistics” option. For the characterization, the death year and the municipality of occurrence were considered to elaborate the “population density” variable, adopting the number of inhabitants for 2010 (approximately half of the period under analysis) and the size of the municipality. The number of inhabitants for 2010 was taken from population projections published by Departamento Administrativo Nacional de Estadística (DANE), Municipal series of the population by area for the period 1985-2017 (freely available online here); the size of the municipality was taken from Municipios de Colombia (freely available online here). Subsequently, based on these data, the population density for each municipality was calculated as follows: population/km ^2^. Subsequently, the quartiles were obtained considering the density of all the municipalities to classify them into municipalities with low, moderate, high, and very high population density based. Mendeley Data: Multiple Correspondence Analysis Syntax for profiling analysis. https://doi.org/10.17632/tj6xkjh5vk.1. ^33^ This project contains the following extended data:
- Sintaxis.docx (main syntaxis to develop multiple correspondence analysis) Data are available under the terms of the Creative Commons Zero “No rights reserved” data waiver (CC0 1.0 Public domain dedication).
