# Supplementary material for: Mortality due to traffic accidents in Colombia: Profiles of pedestrians and cyclists, 1998-2019
Source: F1000Res. 2023 Jul 25;12:360. Originally published 2023 Apr 3. [Version 2] doi: 10.12688/f1000research.131431.2 (PMC10415722; doi:10.12688/f1000research.131431.2)
Supplement: Peer review history

# Peer review: Reviewer response for version 2

**DOI:** 10.5256/f1000research.152870.r192440

**Published:** 2023-08-10

**Authors:** Amir Ali Khan

**Affiliations:** 1 Deenbandhu Chhotu Ram University of Science and Technology, Murthal, Haryana, India

This paper comprehensively studies mortality due to traffic accidents in Colombia. Road accident is the main concern the world is facing. Pedestrians and cyclists are the most vulnerable road users, and there is a considerable need for constructive research on pedestrians and cyclists. This study used multiple correspondence analysis for the identification of profiles of road users. The study design and the investigation is clear and concise. Overall, this study is well-presented and interesting. Pieces of literature used in this study are current, and data sources are reliable. However, there is still a need for some improvements. Authors need to take care of the following suggestions:

- In the Introduction, I suggest including more about the study's objectives.
- I suggest including a graph showing the number of traffic (road) accidents that occurred during 1998-2019 and in how many accidents, pedestrians and cyclists were involved year-wise. Percentage-wise data on accidents involving pedestrians and cyclists will increase the importance of the study. Figure number 10 of the referenced paper ^1^ may be helpful for authors to address this.
- In the discussion section, the authors used too many references. It is suggested to keep references that are most relevant, and the rest of the references can be added in the Introduction.

Is the work clearly and accurately presented and does it cite the current literature?

Yes

If applicable, is the statistical analysis and its interpretation appropriate?

Yes

Are all the source data underlying the results available to ensure full reproducibility?

Yes

Is the study design appropriate and is the work technically sound?

Yes

Are the conclusions drawn adequately supported by the results?

Yes

Are sufficient details of methods and analysis provided to allow replication by others?

Yes

Reviewer Expertise:

Road Safety, Accident Analysis, traffic and transportation engineering

I confirm that I have read this submission and believe that I have an appropriate level of expertise to confirm that it is of an acceptable scientific standard.

## References

[1] : Road Accident Analysis and Safety Audit: A Case Study of MDR 132. *Communications - Scientific letters of the University of Zilina* .2023;25(2) : 10.26552/com.C.2023.026 F26-F38 10.26552/com.C.2023.026

# Peer review: Reviewer response for version 1

**DOI:** 10.5256/f1000research.144273.r169239

**Published:** 2023-05-11

**Authors:** Wilson Giovanni Jiménez Barbosa

**Affiliations:** 1 Universidad Jorge Tadeo Lozano, Bogotá, Colombia

It is very interesting research that addresses a critical problem for Colombian public health.

 The mortality of cyclists and pedestrians generates impacts on the well-being of the population and on the costs of the health system.

 The methodology used is clear and concise. Use reliable and verifiable official sources.

 It is desirable that the results describe the number of bicyclist and pedestrian fatalities for each year in each one of the four defined groups of cities.

 Among the results there is a finding that is not given due relevance: the high percentage of deaths, both cyclists and passers-by without affiliation to the health system. This finding would warrant further analysis to determine its behavior in each year of the period, since the number of people not affiliated with the health system has been constantly decreasing.

 I believe that the description of the profiles of deceased cyclists and passers-by could be presented by comparing the differences in the profiles of deceased between the years 1998 and 2019 for each group of cities. This would give a more specific perspective of the changes in the social characteristics of the deceased during the period.

 I suggest that in the introduction and in the discussion the history of the impulse that has been given to the use of bicycles in the country, particularly in Bogotá, be described as an alternative means of transport that reduces pollution, traffic congestion and that brings health benefits to its users.

Is the work clearly and accurately presented and does it cite the current literature?

Yes

If applicable, is the statistical analysis and its interpretation appropriate?

Yes

Are all the source data underlying the results available to ensure full reproducibility?

Yes

Is the study design appropriate and is the work technically sound?

Yes

Are the conclusions drawn adequately supported by the results?

Yes

Are sufficient details of methods and analysis provided to allow replication by others?

Yes

Reviewer Expertise:

Health care policies

I confirm that I have read this submission and believe that I have an appropriate level of expertise to confirm that it is of an acceptable scientific standard.

# Response

**Published:** 2023-07-09

**Authors:** Maite Catalina Agudelo Cifuentes

**Affiliations:** CES University, Colombia

We appreciate your suggestions to improve the article. In the next lines, we describe each of the adjustments made considering your recommendations:

 1. It is desirable that the results describe the number of bicyclist and pedestrian fatalities for each year in each one of the four defined groups of cities.

**Response:** We agree with the evaluator; we made additional analysis guided to show the behavior of deaths of pedestrians and cyclists by population density for each year analyzed.

 2. Among the results there is a finding that is not given due relevance: the high percentage of

 deaths, both cyclists and passers-by without affiliation to the health system. This finding would warrant further analysis to determine its behavior in each year of the period, since the number of people not affiliated with the health system has been constantly decreasing.

**Response:** We agree with the evaluator; we analyzed the number and percentage of pedestrians and cyclists by health insurance regime. Particularly the number of people uninsurance has decreased by year; between 1998 to 2019, we report a decrease of 96.90%. This behavior is not exclusive to people who died on the roads; according to the data Health Ministry of Colombia is similar to health insurance behavior in the general population of Colombia. 

 3. I believe that the description of the profiles of deceased cyclists and passers-by could be presented by comparing the differences in the profiles of deceased between the years 1998 and 2019 for each group of cities. This would give a more specific perspective of the changes in the social characteristics of the deceased during the period.

**Response:** We agree with the evaluator; it is relevant that profiles highlight the possible differences by population density. In other papers, this relation was reported. Following the literature review that supported the methodology of this article, it was relevant to highlight that mortality by road accidents is a multifactorial phenomenon involving different variables. In this sense, population density is a variable we consider for the profile's construction in pedestrians and cyclists.

 Therefore, we consider it unnecessary to show profiles stratified by population density because this variable is one of the other variables explaining the behavior of mortality by road accidents, which is evidenced in our analysis.

 4. I suggest that in the introduction and in the discussion the history of the impulse that has been given to the use of bicycles in the country, particularly in Bogotá, be described as an alternative means of transport that reduces pollution, traffic congestion, and that brings health benefits to its users.

**Response:** We agree with the evaluator, considering that in this article, we included an analysis of the behavior of cyclists' mortality, and recently, the bicycle, as a means of transport, has been promoted as an alternative to motor vehicles; we included some contextual data on the development of bicycle infrastructure and their use in our country.
